# Lighting the Path: Raman Spectroscopy’s Journey Through the Microbial Maze

**DOI:** 10.3390/molecules29245956

**Published:** 2024-12-17

**Authors:** Markus Salbreiter, Sandra Baaba Frempong, Sabrina Even, Annette Wagenhaus, Sophie Girnus, Petra Rösch, Jürgen Popp

**Affiliations:** 1 Institute of Physical Chemistry and Abbe Center of Photonics, Friedrich Schiller University, Helmholtzweg 4, 07743 Jena, Germany; markus.salbreiter@uni-jena.de (M.S.); sandra.frempong@uni-jena.de (S.B.F.); juergen.popp@uni-jena.de (J.P.); 2 InfectoGnostics Research Campus Jena, Center of Applied Research, Philosophenweg 7, 07743 Jena, Germany; 3 Leibniz-Institute of Photonic Technology, Member of the Leibniz Research Alliance—Leibniz Health Technologies, Albert-Einstein-Str. 9, 07745 Jena, Germany; 4 Cluster of Excellence Balance of the Microverse, Friedrich Schiller University Jena, 07743 Jena, Germany

**Keywords:** Raman spectroscopy, bacteria identification, excitation wavelength, single cell analysis, bulk analysis, Raman fiber probe

## Abstract

The rapid and precise identification of microorganisms is essential in environmental science, pharmaceuticals, food safety, and medical diagnostics. Raman spectroscopy, valued for its ability to provide detailed chemical and structural information, has gained significant traction in these fields, especially with the adoption of various excitation wavelengths and tailored optical setups. The choice of wavelength and setup in Raman spectroscopy is influenced by factors such as applicability, cost, and whether bulk or single-cell analysis is performed, each impacting sensitivity and specificity in bacterial detection. In this study, we investigate the potential of different excitation wavelengths for bacterial identification, utilizing a mock culture composed of six bacterial species: three Gram-positive (*S. warneri*, *S. cohnii*, and *E. malodoratus*) and three Gram-negative (*P. stutzeri*, *K. terrigena*, and *E. coli*). To improve bacterial classification, we applied machine learning models to analyze and extract unique spectral features from Raman data. The results indicate that the choice of excitation wavelength significantly influences the bacterial spectra obtained, thereby impacting the accuracy and effectiveness of the subsequent classification results.

## 1. Introduction

The identification of bacteria, fungi, viruses, and other microorganisms has traditionally been based on gold standard methods such as culture-based techniques and genetic analysis tools. However, these methods can be complex and present various challenges. While they allow for the direct or indirect detection of pathogens, they differ in speed, precision, and cost, each offering distinct advantages and drawbacks. For bacterial and fungal infections, plating techniques paired with automated devices for species identification and antibiotic susceptibility testing (AST) are commonly used and typically provide accurate identification [1,2]. However, these methods are slow, often requiring several days to produce results, which is a significant drawback, especially in cases of acute, life-threatening infections. This limitation can be addressed by PCR-based techniques, which deliver accurate results within hours, but their high cost limits their use in many hospital and environmental settings. In addition, most of these techniques are prone to human error and are time consuming since they need trained personnel that can not only apply these techniques but use them effectively to detect the pathogens within the sample as well as identify them based on certain criteria [3,4,5,6,7,8].

It is therefore clear that alternative methods that are not only cheaper and faster but deliver equally accurate results need to be employed. One such alternative method is Raman spectroscopy [9,10,11,12,13]. Raman spectroscopy (RS) has become a key technique for identifying bacterial species by analyzing their molecular structures and behaviors. The Raman effect, first described by C.V. Raman in 1928 [14,15,16,17], involves the inelastic scattering of photons, where the energy of the scattered photon is either lower (Stokes) or higher (anti-Stokes) than the incoming photon [18,19,20]. This energy shift, known as the Raman shift, corresponds to the vibrational frequencies of molecular components, such as proteins, lipids, and nucleic acids within the bacteria [9,10,11,12,13,21,22,23,24]. By generating a vibrational spectrum that reflects the unique molecular signature of a bacterium, Raman spectroscopy allows for the precise identification and differentiation of bacterial strains, making it a valuable tool in microbial diagnostics and research [25,26,27].

The efficiency of the Raman scattering process relies on the fourth power of the frequencies of the excitation photons [28,29]. Since the Raman effect is strongly influenced by the excitation frequency, it is inherently more pronounced in the ultraviolet (UV) region and weaker in the infrared. When UV light is used for excitation, the intensity of scattered photons increases significantly. If the energy of the incident photon matches the electronic transition of a molecule, a resonance effect occurs, further amplifying the Raman signal. Molecules in resonance experience a much larger increase in intensity compared to those that are not, making this effect particularly useful for enhancing the detection of specific molecular features [28,30,31].

Various Raman spectroscopic techniques have been developed for analyzing biological samples, including cells, bacteria, and tissues [32,33,34,35,36,37,38,39,40,41,42,43,44,45,46,47]. When combined with a microscope, a Raman setup can resolve structures as small as 1 µm, enabling the direct measurement and identification of single bacterial cells without the need for cultivation, thus accelerating the identification process. Although, for this method, isolation steps are required, they can be completed in under two hours. Additionally, Raman microscopy can examine subcellular structures in eukaryotic cells, facilitating the study of intracellular bacteria [48,49,50,51,52]. Raman microscopy is useful for bacterial analysis due to the complexity and sensitivity of bacterial Raman spectra. It enables the precise identification of species by resolving overlapping spectral features and distinguishing biochemical signatures, such as proteins, lipids, and nucleic acids. This level of resolution is essential for detecting small chemical variations in bacterial cells, such as those related to metabolism or antibiotic resistance. Furthermore, high spectral resolution facilitates the accurate analysis of bacteria in complex, multicomponent environments, making it invaluable for rapid, non-destructive diagnostic applications [39,42,53,54,55,56,57].

In bacterial analysis, the primary differences among these methods lie in the choice of excitation wavelength and whether bulk or single-cell analysis is performed. Different wavelengths reveal distinct profiles of bacterial biochemical composition, depending on how close the excitation wavelength is to the absorption spectrum of the biomolecules. For single-cell analysis, not all wavelengths are suitable—UV light can cause photothermal damage, while near-infrared (NIR) light may produce weak signals. Additionally, cell-to-cell variability due to differences in metabolic and cell cycle stages can introduce spectral variations, potentially reducing classification accuracy. However, single-cell analysis requires minimal biomass, allowing for faster—or no—incubation and analysis. Nevertheless, single-cell analysis always requires an isolation step prior to analysis. In contrast, bulk analysis, measuring thousands of cells at once, averages out sample heterogeneity, improving the signal-to-noise ratio, with the drawback being longer incubation times to obtain sufficient biomass for analysis. Nevertheless, preliminary cultivation steps standardize the workflow and improve consistency [25,58,59]. With the growing need for rapid and accurate bacterial identification, Raman spectroscopy has become an increasingly important technique, utilizing systems with a range of excitation wavelengths across the UV, visible, and infrared spectrum.

UV resonance Raman (UVRR) spectroscopy is commonly used to selectively enhance the Raman signal from chromophores, as most molecules have absorption bands in the UV region. A key advantage of UVRR is that it requires no chemical labelling or modification of the molecule under study. Bacteria, bacterial spores, viruses, and cyanobacteria produce high-quality UV-excited resonance Raman spectra for two main reasons: a strong absorbance of taxonomically significant components in the UV region and the absence of protein fluorescence below 260 nm [60,61,62,63]. Fluorescence-free resonance Raman spectra, especially between 190 and 250 nm, enable detection even from highly fluorescent bacteria like *Pseudomonas* spp. which secrete fluorescing siderophores called pyoverdine and pyocyanin [9]. Excitation at different UV wavelengths (e.g., 222, 231, 242, and 251 nm) highlights distinct contributions from nucleic acids and proteins. Spectral changes are influenced by the absorption of bacterial components, and intermolecular interactions such as nucleic acid base-pair stacking affect spectral intensity. The “internal filter effect” may cause weak nucleic acid scattering due to strong absorption by proteins in outer bacterial membranes, especially at higher frequencies [64,65].

If the Raman spectra of bacteria are excited at 244 [56,59,62,63,66,67,68,69,70,71,72,73,74,75] or 257 nm [62,66,73,74,75], the resonance Raman spectra primarily reveal nucleic acid purine and pyrimidine ring contributions. Using 228 nm excitation is especially useful for protein analysis, as it selectively highlights the vibrational spectra of aromatic residues while minimizing interference from other residues, the protein backbone, or buffer components. Here, the most prominent Raman signals derive from aromatic amino acids such as tryptophan, tyrosine, and phenylalanine [76,77,78,79,80,81]. At lower wavelengths, weak nucleic acid peaks reappear along with predominately strong amino acid contributions. This makes UVRR an effective tool for detailed amino acid [60,61,76,77,78,79,80,81,82,83,84,85,86,87,88,89,90,91,92,93,94,95,96,97,98,99,100,101,102,103,104,105,106,107,108], nucleic acid [60,61,89,102,103,105,106,107,108,109,110,111,112,113,114], protein [115,116,117,118,119,120,121,122,123,124], and bacterial [56,59,60,62,63,66,67,68,69,70,71,72,73,74,75,107,108,125,126] studies among other applications [127,128,129,130,131,132,133,134,135].

Lasers in the visible (532 and 633 nm) and near-infrared (>785 nm) ranges are essential for effective cellular and microbial analysis, balancing spatial resolution, autofluorescence avoidance, and optimal penetration depth with minimal photodamage depending on the laser excitation wavelength selection. Visible wavelengths, particularly 532 nm, are often ideal for high-resolution, single-cell analysis, allowing the precise targeting of individual cells through Raman microscopy. This approach allows for the detailed examination of individual cells, revealing insights into the heterogeneity and functional diversity among microbial populations that are often obscured in bulk measurements [136,137,138]. While visible lasers are widely recognized for their effectiveness in Raman spectroscopic single-cell analysis, they are also applied for bulk measurements, enabling a comprehensive characterization of cellular populations and their collective properties [139,140,141,142,143,144]. Still, direct analysis from real-world matrices presents challenges, as chemical variations in both bacteria and sample matrices—such as blood, urine, or wastewater—can affect the Raman spectra. Consequently, single-cell analysis databases must be extensive, capturing a broader range of matrix variations and environmental influences beyond what is typically required for bulk samples [58,71,145]. An effective approach to enhance data reliability involves producing and measuring multiple replicates of each strain or species, allowing for a more comprehensive analysis [58]. In certain cases, particularly in culture-dependent analyses, pre-cultivation steps may be necessary. This often involves cultivating bacteria on agar plates, in liquid media, or in simulated environments that mimic real-world conditions [58,140,146,147,148,149,150,151]. Here, it is crucial to maintain culture parameters closely aligned with real-world conditions, though achieving complete accuracy is challenging under laboratory settings [58]. Various Raman-compatible isolation techniques—such as density gradient centrifugation, enzymatic matrix digestion, culture-independent filtration, dielectrophoresis, and antibody capture—have proven effective as isolation steps across different sample types [145,146,147,150,151,152,153], making them useful for both culture and culture-independent analysis. Despite the inherent complexities of these methods, the accurate identification of single bacterial cells can be accomplished in as little as below two hours for culture-independent analysis [154,155,156,157], or within 24 h or more for culture-dependent analysis.

Single-cell analysis in bacterial research has a range of applications, including the study of food quality control [142,143,145], antibiotic resistance mechanisms [59,140,158,159,160,161], and bacterial metabolic variability at the different growth phases among cells in mixed populations [153,162,163,164,165,166,167,168]. Additionally, this method can be used to analyze gene expression patterns at the single-cell level [138,169,170,171], or spore germination dynamics [172,173,174,175]. By exploiting the resonance Raman effect at 532 nm, biomarker molecules present at low concentrations—such as carotenoids [176,177,178] or cytochromes [179,180,181,182,183,184,185]—can be monitored effectively in bacterial cells. Even in the absence of these biomarkers, metabolic activities or microbial interactions with the environment can still be studied by incorporating isotopically labelled substrates (stable isotope labelling, SIP) into bacterial cells, allowing the detection of actively incorporating microbes [137,137,154,186,187,188,189,190,191,192,193].

However, while the 532 nm wavelength is advantageous for tracking these specific biomolecular interactions, it also induces significant autofluorescence in bacterial samples. This property, although challenging for Raman analysis, becomes highly beneficial in fluorescence-based techniques such as fluorescence microscopy and flow cytometry [154,194]. When used in Raman microscopy alongside fluorescence staining, the 532 nm laser effectively excites both natural autofluorescent molecules and targeted fluorescent dyes, producing high-resolution images that reveal intricate bacterial cell structures, protein localization, and intracellular dynamics [48,49,50,51,52,195,196,197]. This dual capability makes 532 nm excitation particularly valuable, supporting both detailed biochemical monitoring and high-contrast imaging in studies where cellular structure and native activity are central to the analysis.

Meanwhile, the NIR wavelength region is regarded as highly versatile, being used not only for single-cell analysis [198,199] but also for examining bacterial colonies. At this wavelength, either a confocal Raman microscope for detailed imaging [40,140,143,200,201,202,203,204,205,206,207,208,209,210,211] or a fiber probe [59,136,144,212,213,214] is used for spectra acquisition, allowing for flexibility based on the sample type and specific analytical requirements. However, as shown by Puppels and colleagues, the advantage of confocal microscopy over fiber-optic probes at this excitation wavelength is particularly evident for small sample volumes. Confocal Raman microspectroscopy enables precise measurements within volumes as small as microcolonies, making it especially valuable for detailed analysis at the microscale [202,215]. The 785 nm laser excites the overall cellular matrix, capturing broad molecular information. For example, staphyloxanthin—a carotenoid pigment from *Staphylococcus aureus*—can be detected via Raman spectroscopy, although its signal is often less intense than that from shorter wavelengths like 532 nm. Nevertheless, excitation at 785 nm most often omits photobleaching [59,140,209,211,212,216,217,218]. Overall, this wavelength finds applications across various bacterial studies, including the examination of bacteria within tissues or biofilms, metabolic profiling, and exploring antibiotic resistance mechanisms and antibacterial studies, among others [59,140,199,206,211].

To our knowledge, no comprehensive studies have systematically examined how different excitation wavelengths impact spectral outcomes critical for reliable bacterial classification. This study aims to fill this gap by providing an in-depth analysis of the effects of various excitation wavelengths on spectral data, exploring their potential to enhance accuracy and reliability in bacterial identification. To illustrate this, we used six different bacteria in a mock culture, comprising three Gram-positive species (*Staphylococcus warneri*, *Staphylococcus cohnii*, and *Enterococcus malodoratus*) and three Gram-negative species (*Pseudomonas stutzeri*, *Klebsiella terrigena*, and *Escherichia coli*). Nevertheless, *K. terrigena* and *E. coli* as well as the two staphylococci (*S. warneri* and *S. cohnii*) exhibit very similar spectral patterns making it sometimes difficult to distinguish between them. To address this challenge, we employed machine learning models to extract the distinctive features of each species and facilitate their classification. The effectiveness of our model was validated using an independent validation replicate, further enhancing the robustness of our findings. The results revealed that machine learning holds significant promise for the rapid and accurate discrimination of bacterial species, even those that are closely related. This emphasizes the potential of Raman spectroscopy not just as a diagnostic tool, but as a transformative approach in microbial identification [219,220].

## 2. Results and Discussion

### 2.1. UV Resonance Raman Spectroscopy (UVRR)

**Figure 1:**
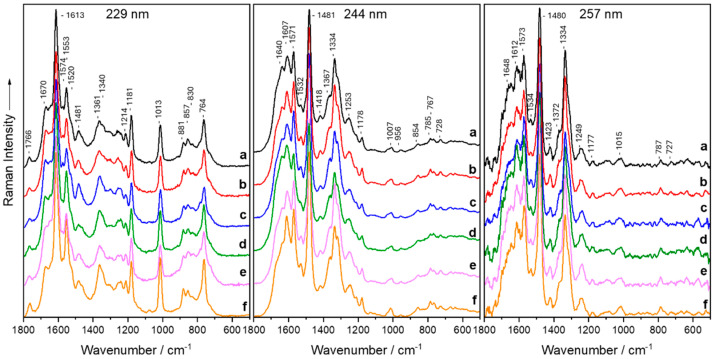
Mean UV resonance Raman spectra of *S. warneri* (**a**), *S. cohnii* (**b**), *P. stutzeri* (**c**), *K. terrigena* (**d**), *E. malodoratus* (**e**), and *E. coli* (**f**) measured at 229, 244, and 257 nm.

For our UVRR study, three different excitation wavelengths (229, 244, and 257 nm) were used whereby 60 spectra for each bacterium were obtained from three biological replicates. The UVRR mean spectra are shown for comparison in Figure 1. These three excitation wavelengths provide a clear indication of the distinctive UVRR fingerprints of the six bacteria. For each excitation wavelength, the UVRR spectra of the six investigated species are very similar and do not reveal huge variances. In the UVRR spectra, the signals from nucleic acid bases and aromatic amino acids are amplified for the excitation wavelengths 244 and 257 nm, whereas the excitation with 229 nm specifically excites protein moieties. This allows differences to be captured at the genetic/transcriptional level and partly on the protein level.

The most prominent bands of the bacterial UVRR spectra are assigned by comparing those with the UVRR spectra of well-known constituents. Since the DNA/RNA and protein components (aromatic amino acids) are enhanced in the deep UV region, the signal assignment will be focused on these components. In Figure 1, the 229 nm excitation wavelength gives rise to bands from tryptophan at 764, 857, 881, 1013, 1340, 1361, 1520, 1553, and 1766 cm^−1^ [79,84,106,221,222,223,224,225] as well as tyrosine at 830, 1181, 1214, and 1613 cm^−1^ [79,82,84,221,225,226]. The band at 1481 cm^−1^ derives mainly from guanine and adenine C8H deformation and N9C8 and C8N7 stretching vibrations [106,224,226,227], whereas 1670 cm^−1^ indicates the amide I band [228,229].

The excitation at 244 and 257 nm displayed obvious differences in the signal-to-noise ratio and the increased background of the 257 nm spectra. These differences can be attributed to a beginning fluorescence influence of the fused silica slides and the investigated bacteria at 257 nm. The main UVRR signals can be mainly assigned to proteins and DNA/RNA components (Figure 1). A detailed band assignment is shown in Table 1.

The vibrations deriving from aromatic amino acids like phenylalanine, tyrosine, and tryptophan can be found at 764, 854, 956, 1007, and 1178 cm^−1^ [79,82,84,221,222,223,224,225,226] for 244 nm, while 257 nm displays them at 1015, 1177, and 1612 cm^−1^ [82,84,221,224,225,226]. The Raman signals of the DNA/RNA bases (adenine, thymine, cytosine, and guanine) can be observed at 728, 785, 1178, 1334, 1367, 1418, 1481, 1532, 1571, and 1640 cm^−1^ [72,82,101,106,158,163,221,222,224,225,226,227,230,231] at 244 nm. The amide III signal can be detected at 1253 (244 nm) and 1249 cm^−1^ (257 nm) [222].

**Figure 2:**
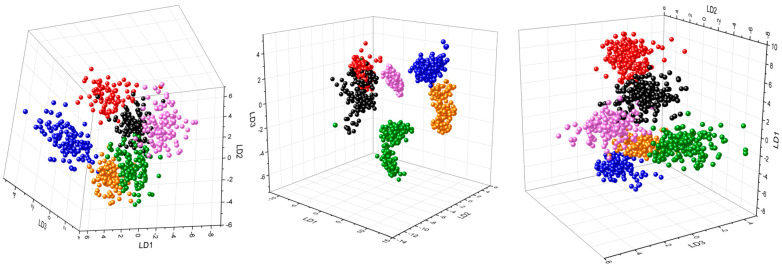
PCA–LDA results of classification of bacteria measured at 229 nm (**left**), 244 nm (**middle**), and 257 nm (**right**). *S. warneri* (black), *S. cohnii* (red), *P. stutzeri* (blue), *K. terrigena* (green), *E. malodoratus* (magenta), and *E. coli* (orange).

Since the Raman spectra are very similar within their respective excitation wavelengths, machine learning analysis was necessary for differentiating them (Figure 2 and Table 2 and Table 3). To highlight small variations in the Raman spectra, chemometric methods must be used. In this case, a combination of principal component analysis and a linear discriminant analysis (PCA–LDA) was chosen. Here, PCA is used for dimensionality reduction by projecting the data onto only a few principal components (PCs) while preserving as much of the data’s variation as possible. A linear discriminant analysis (LDA) model was created and validated by performing N-fold cross validation with varying optimal PCs for each excitation wavelength. The ideal number of PCs for each model was optimized by finding the best cross validation on the training dataset based on the mean accuracy of the trained LDA model. To minimize the risk of overfitting, a PCA–LDA model was selected, as only six bacterial classes were chosen, and a limited number of Raman spectra were measured. Alternative models, such as support vector machines (SVMs) or convolutional neural networks (CNNs), were not utilized due to their higher susceptibility to overfitting with small datasets. The validation of the training set was performed by using an independent batch of the mock culture. Each dot in Figure 2 represents a single spectrum.

Table 2 and Table 3 summarize the classification and validation outcomes. An accuracy of 98.1% was achieved by correctly classifying 653 out of 666 Raman spectra collected at 229 nm. Comparable accuracies were obtained for the excitation wavelengths of 257 nm and 244 nm, with accuracies of 97.6% (1032/1057) and 96.1% (992/1032), respectively. Misclassifications primarily occurred between the closely related *S. warneri* and *S. cohnii*, or within the Gram-positive group. The LDA plots (Figure 2) for all three excitation wavelengths highlight the misclassifications between the two *Staphylococcus* species, with the 244 nm wavelength exhibiting more misclassifications than the 229 nm and 257 nm wavelengths. Generally, most errors involved classifying *S. warneri* as *S. cohnii* rather than the reverse. Despite their close relationship, *K. terrigena* and *E. coli* were relatively well-separated in all LDA plots. However, 18 out of 180 (10%) Raman spectra of *E. malodoratus* were incorrectly identified as *S. warneri* in the LDA plot at 257 nm.

To evaluate the predictive capability of the classification model, a validation process was conducted using an independent replicate of the mock culture. The bacterial strains in this independent replicate were cultivated and measured under identical conditions. To assess the sensitivity of the validation procedure, the classification model was subsequently employed to predict the outcomes for these independent test datasets. In Table 3, the validation accuracies of all three models ranged from 76.7% to 89.7%, with each model exhibiting significant misclassifications. Specifically, 40% (24/36) of *K. terrigena* and 13% of *P. stutzeri* Raman spectra were incorrectly identified as *E. coli* during the validation of the 229 nm data. Conversely, the two *Staphylococcus* species were effectively distinguished from each other. However, during the 244 nm validation, 75% (45/60) of *S. warneri* spectra were misclassified as *S. cohnii*, though the reverse misclassification did not occur. This result reflects the training outcome of the classification (Table 2). In the 257 nm validation, similar misclassifications were observed, with *S. warneri* spectra being misclassified as *S. cohnii* (18/60) and *E. malodoratus* spectra as *S. cohnii* (47/60). Notably, these misclassifications were confined to the Gram-positive bacteria. These findings suggest that the LDA models developed can validate independent test data to a certain extent.

### 2.2. Raman Microscopy

**Figure 3:**
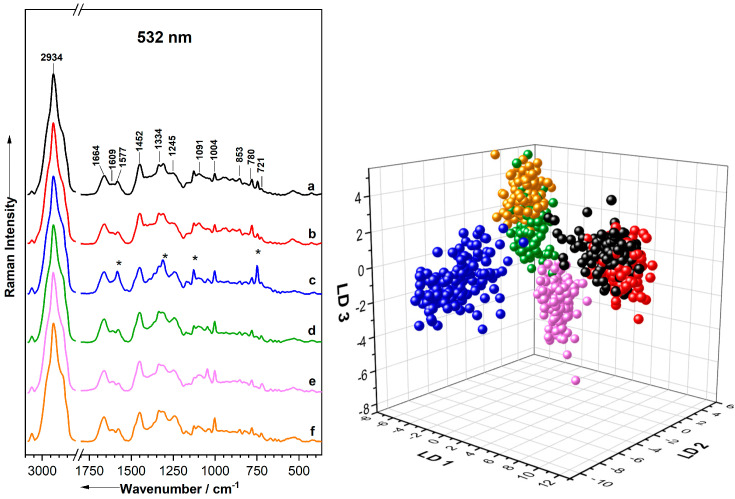
Mean Raman spectra (**left**) from a Raman microscope with 532 nm excitation and the corresponding PCA–LDA results of classification (**right**) of *S. warneri* (a, black), *S. cohnii* (b, red), *P. stutzeri* (c, blue), *K. terrigena* (d, green), *E. malodoratus* (e, magenta), and *E. coli* (f, orange). *: cytochrome.

In the Raman microscopy study, a single-cell analysis approach was performed using 532 nm excitation whereby ~200 spectra for each bacterium species were obtained from four biological replicates. The mean spectra are presented in Figure 3. The resulting Raman spectra reveal a range of vibrational signatures corresponding to the amount of the molecular components found in the bacterial cells [162,232,233].

Key spectral features include distinct peaks associated with nucleic acids, such as DNA and RNA, with prominent bands around 1577, 1095, 785, and 724 cm^−1^ [72,158,163,186,207,222]. Proteins are represented by characteristic amide I and amide III vibrations near 1664 and 1245 cm^−1^ [59,136,207], respectively, the C=C ring vibrations of phenylalanine and tyrosine at 1605 cm^−1^, the phenylalanine ring breathing vibration at 1004 cm^−1^, and the ring breathing vibration of tyrosine at 854 cm^−1^ [59,136,186]. In some cases, peaks result from the combined contributions of different molecular components. For example, the signal at 1448 cm^−1^ is a result of the deformation vibrations of the CH_2_ and CH_3_ groups commonly found in lipids, proteins, and carbohydrates [59,228,234,235]. Similarly, the signal at 2934 cm^−1^ is as a result of the C-H stretching vibrations of the CH_3_/CH_2_/CH groups found in the proteins, lipids, nucleic acids, and carbohydrates [59,186,229,236]. Additionally, bacteria contain chromophores such as carotenoids and cytochromes which exhibit strong resonance effects, particularly at an excitation wavelength of 532 nm [56,237,238]. This can cause a strong enhancement of these molecules, especially in pigment-containing bacteria. For instance, in the spectra of *P. stutzeri*, intense signals observed at 1583, 1312, 1126, and 749 cm^−1^ can be attributed to cytochrome vibrations (*) [72,140,185,237,239,240,241,242,243]. A detailed band assignment is shown in Table 4.

For classification, a PCA–LDA model was utilized, and the results are summarized in Table 5. The model achieved an overall classification accuracy of 95%, correctly identifying 1015 out of 1069 Raman spectra. However, misclassifications were observed, particularly between closely related species, such as *S. warneri* and *S. cohnii*, as well as *K. terrigena* and *E. coli*, or within the larger Gram-positive and Gram-negative groups. For instance, 15 out of 190 spectra of *K. terrigena* were wrongly classified as *E. coli*, while six were misclassified as *E. malodoratus*. Similarly, seven out of one hundred eighty-nine spectra of *E. coli* were misclassified as *K. terrigena*. In the case of *S. warneri*, 14 out of 172 spectra were misclassified as *S. cohnii*, with one spectrum also being incorrectly classified as *K. terrigena.* Nevertheless, the highest classification accuracy was achieved for *E. malodoratus* and *P. stutzeri*. These patterns can be further visualized in the LDA plot in Figure 3 (right), where each dot represents an individual Raman spectrum of one single bacterial cell corresponding to a specific species.

The classification model was validated using an independent replicate of mock cultures, achieving an accuracy of 89.3% (Table 5) by correctly identifying 268 out of 301 Raman spectra. Misclassifications were noted both among closely related species and within unrelated species. For instance, 20% (10/50) of *K. terrigena* spectra were misclassified as *E. coli*. Conversely, 34.6% (18/52) of *S. warneri* were misclassified as *K. terrigena*, and 3.7% (2/54) were misclassified as *S. cohnii*. Additionally, 2.2% (1/46) of *P. stutzeri* were incorrectly identified as *E. coli*, while 1.8% (1/53) of *S. cohnii* were misidentified as *K. terrigena* and *E. malodoratus*, respectively. These findings suggest that while LDA models can offer valuable insights and partial validation of independent data, misclassifications are a significant challenge. They often arise from overlapping spectral features among closely related species, as well as similarities in biochemical properties among unrelated species, leading to confusion in the classification process. Additionally, misclassifications may reflect inherent biological variability within the single cells, further complicating accurate identification.

### 2.3. Raman Fiber Probe

**Figure 4:**
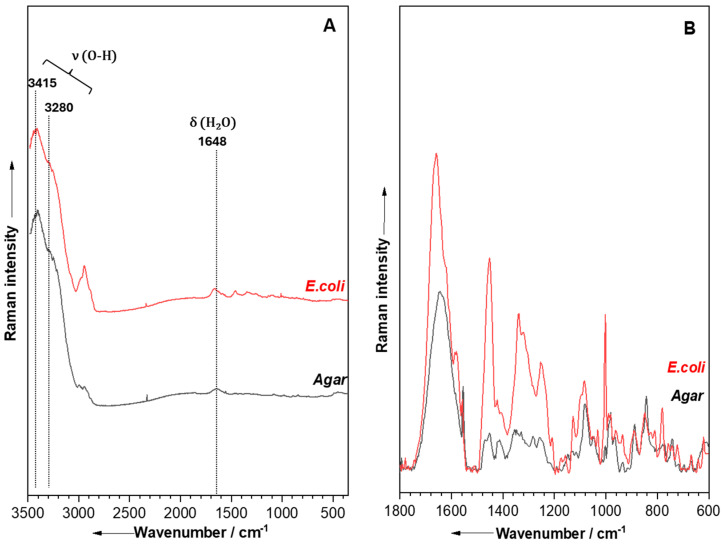
Raman spectra measured with 785 nm excitation of an *E. coli* colony (red) and agar (black): (**A**) in the region 3100−350 cm^−1^ (raw data) before baseline correction, and (**B**) in the region 1800−500 cm^−1^ after baseline correction. ν = stretching modes, δ = bending modes.

In contrast to Raman microscopy, where spectra from single bacterial cells were measured, this section discusses the spectra obtained from bacterial colonies directly on an agar plate using a Raman fiber probe (FP-RS) with 785 nm excitation. Approximately 60 spectra were collected for each bacterial species from three biological replicates. However, the considerable depth of field of the fiber probe enables it to capture signals not only from the bacterial colonies but also from the underlying culture medium which can lead to overlapping spectra or strong fluorescence [202,212,247,248]. While this interference from the agar is relatively minimal compared to the stronger signals from the bacterial colonies, variations in the thickness of these colonies can affect the agar’s contribution to the overall Raman spectra collected from colonies [202,212]. Another major challenge in this analysis is the strong interference from the various O–H vibration modes of water molecules in the agar (Figure 4) [230,249,250,251], which complicates the process further. Therefore, signals above 2000 cm^−1^ are generally excluded during 785 nm excitation due to their low intensity [212].

**Figure 5:**
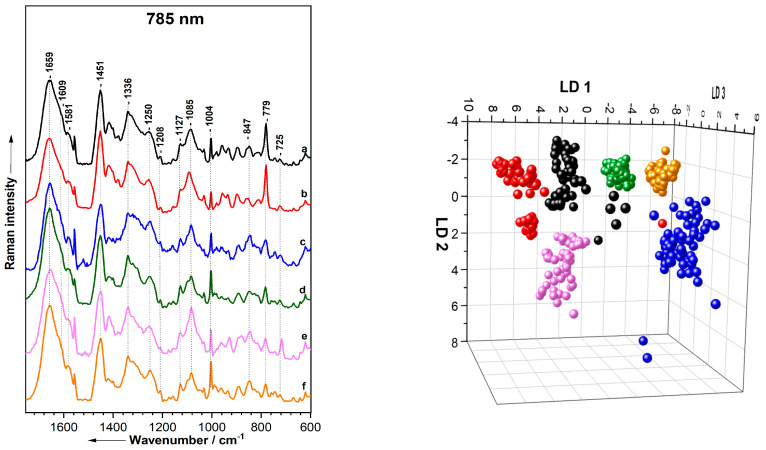
Mean Raman spectra (**left**) from a Raman fiber probe with 785 nm excitation and the corresponding PCA–LDA results of classification (**right**) of *S. warneri* (a, black), *S. cohnii* (b, red), *P. stutzeri* (c, blue), *K. terrigena* (d, green), *E. malodoratus* (e, magenta), and *E. coli* (f, orange).

As a result, only the fingerprint range (1800–600 cm^−1^) is displayed in the spectra obtained using the FP-RS. The mean Raman spectra for the bacterial species are presented in Figure 5 (left), while Table 6 details the significant Raman bands along with their tentative assignments.

Aside from the wavenumber range, the Raman spectra obtained from FP-RS exhibit characteristic peaks similar to those from the Raman microscope system. Notable bands include nucleic acids, represented by peaks at 1581, 1336, 1085, 779, and 725 cm^−1^, and protein signals with bands at 1659, 1609, 1250, 1208, 1004, and 847 cm^−1^. Additional features in the FP-RS spectra include the symmetric stretching vibrations of the C–O–C glycosidic linkage for carbohydrates and the C–C stretching vibrations of unsaturated fatty acids in the lipid band at 1127 cm^−1^.

As shown in Table 7, PCA–LDA was employed to distinguish between the bacterial species. The model achieved an overall classification accuracy of 98.7%, successfully classifying 370 out of 375 Raman spectra. Only a few misclassifications were observed: specifically, one out of fifty-six spectra of *S. warneri* were incorrectly classified as *P. stutzeri*, and one out of sixty-two spectra of *S. cohnii* were also misclassified as *S. warneri*. Furthermore, four out of sixty-five spectra of *P. stutzeri* were misclassified as *E. coli*, *S. cohnii*, *S. warneri*, and *E. malodoratus*. The high classification accuracy achieved in bacterial studies can be attributed to several key factors, including the nature of the sample, measurement parameters—particularly excitation wavelength and laser power—and the sensitivity of the instrument. Moreover, each spectrum represents a sum spectrum over a complete colony, capturing not only biochemical information from the bacterial cells but also the contributions of extracellular materials and the diverse metabolic activities within the colony [136,265]. This averaging effect facilitates the identification of consistent spectral patterns associated with specific bacterial colonies, in contrast to the heterogeneity observed at the single-cell level, resulting in higher classification accuracy. The observed patterns are further illustrated in the LDA plot in Figure 5 (right), where each dot represents an individual spectrum corresponding to a specific species.

The classification model was validated using an independent replicate of bacteria, achieving 95.2% accuracy (Table 7), correctly identifying 256 out of 269 Raman spectra. Significant misidentifications occurred within the *Staphylococcus* species, with 6.6% (3/45) of *S. cohnii* spectra misidentified as *S. warneri* and 20% (9/45) of *S. warneri* spectra misidentified as *S. cohnii*. Additionally, 2.3% (1/44) of *P. stutzeri* spectra were misidentified as *E. coli*. These findings suggest that while LDA models can partially validate independent data, difficulties remain in distinguishing closely related species.

### 2.4. Comparison of Excitation Wavelengths

When comparing different excitation wavelengths in Raman spectroscopy, it is essential to first consider the experimental design and define the specific research questions to be addressed. A structured selection process should guide the choice of experimental parameters. For instance, it is critical to determine whether the goal is to obtain a comprehensive profile of the bacterial sample, capturing a broad range of biochemical molecules, or to focus on a targeted subset of biomolecules. Additional considerations include the choice of excitation wavelength, whether to conduct single-cell or bulk measurements, the required sample size, and the optimal Raman spectroscopy setup. Addressing these questions beforehand is crucial, as each decision in the selection process can significantly impact the resulting Raman spectra and, consequently, the interpretations of the experimental findings.

UV resonance Raman (UVRR) spectroscopy is a very powerful tool for selectively investigating the structure of biological molecules, such as aromatic amino acids and nucleic acids. In non-resonant Raman spectroscopy, vibrations from all parts of a molecule contribute to the spectrum with relatively uniform intensities. In contrast, resonance Raman spectroscopy highlights a specific subset of vibrations associated with the resonant chromophore, which dominate the UVRR spectrum. This enhancement makes UVRR highly sensitive and selective, making it particularly well-suited for the study of macromolecules with complex and crowded non-resonant Raman spectra that are otherwise challenging to interpret [62,72,225,258,266,267,268,269,270].

An important advantage of utilizing deep ultraviolet (UV) wavelengths (<260 nm) in Raman spectroscopy is the ability to acquire spectra in a fluorescence-free region while concurrently benefiting from resonance Raman effects. This makes ultraviolet resonance Raman (UVRR) spectroscopy a promising tool for the identification of bacterial species and strains. Yet, it is particularly challenging for small-volume samples and limits the potential for microscopic observations [271]. However, due to photodegradation, UVRR spectra are typically collected from dried bacterial films or suspensions, necessitating substantial biomass for bulk measurements. Photodamage primarily results from strong absorption, where photon energy is sufficient to break excited molecules or prompt reactions with the surrounding medium [82,272,273,274,275,276,277,278,279]. Consequently, it is essential to understand the molecular degradation processes in bacterial cells during UVRR measurements and to carefully control light intensities and exposure times to mitigate these effects [279].

In contrast, both visible and near-infrared lasers for microbial Raman studies offer distinct advantages and limitations based on their interactions with pathogens. Visible lasers, typically in the 400–700 nm range, are commonly used due to their high quantum yield and well-established protocols for Raman spectroscopy [280]. The intensity of the Raman signal is inversely proportional to the fourth order of the wavelength of the excitation light; thus, the 532 nm laser, in the green region of the visible spectrum, often produces stronger Raman signals due to its higher frequency, which enhances scattering efficiency [281,282]. This heightened sensitivity enables more detailed chemical insights into bacterial cell components, such as nucleic acids, proteins, and lipids. Additionally, using a 532 nm laser with a high NA objective achieves a theoretical spot size of less than 1 µm, offering exceptionally high spatial resolution ideal for precise applications, such as single-cell analysis [283,284,285]. Nevertheless, the increased scattering and absorption at this wavelength limits its penetration depth in microbial cells, which can be a drawback. Additionally, the high energy of visible lasers in combination with the small spot size raises the risk of photochemical damage and localized heating, which is problematic for delicate biological samples like bacteria [285,286,287].

Another challenge with visible lasers, especially around 500 nm, is the increased likelihood of autofluorescence from biological materials like pigments or cellular components [154,281,283,288]. This background fluorescence can interfere with the Raman signal, making data interpretation more difficult. Despite these challenges, by focusing on single bacterial cells, the issue of fluorescence can sometimes be mitigated. Furthermore, the capability to analyze single cells can reduce the time needed to observe bacterial responses, as fewer cells are required, eliminating the need for a pre-cultivation step [136,199]. However, a limitation of single-cell measurements is the variability introduced by cell-to-cell differences within heterogeneous populations [59,136,203]. Additionally, due to the resonance Raman enhancement effect, the vibrational modes of molecules such as carotenoids or cytochromes are often intensified, and even at low concentrations, their characteristic Raman signals may dominate the observed spectrum [140,159,185,237,239,240,241,242,243,280]. These factors can lead to a lower signal-to-noise ratio (SNR), further complicating data analysis [283].

On the other hand, 785 nm excitation in the near-infrared (750–1000 nm) range generates generally weaker Raman signals due to the lower frequency, which reduces scattering efficiency. As a result, when comparing the output of UV and visible lasers to that of NIR lasers, the intensity of the spectrum from the NIR laser can be roughly 15 times less intense. Since spatial resolution is inversely proportional to the excitation wavelength, the spatial resolution at 785 nm is lower than that of visible or UV lasers, even for instruments with similar configurations and optics [283,285]. However, a 785 nm confocal microscope achieves higher spatial resolution, with a spot size of approximately 1.1 µm, compared to a 785 nm fiber probe, which typically has a larger spot size in the tens of microns (depending on the probe’s core diameter and NA). This is due to the advanced optical design of the confocal microscope, which includes high NA objective lenses and a confocal pinhole that blocks out-of-focus light, ensuring only the in-focus light reaches the detector [215,285]. This setup allows for more precise focusing and better beam quality compared to fiber probes, which often have lower NA and suffer from beam divergence.

Nonetheless, generally, the 785 nm laser is often preferred for its ability to minimize autofluorescence in biological samples, which can mask the weaker Raman signals produced with visible light excitation [154,202,212,280,281,283]. Also, the lower energy of the incident photons at 785 nm makes it less likely to excite electronic transitions that lead to fluorescence. As a result, this wavelength produces a significantly lower fluorescence background, enhancing the detection of Raman signals and improving the signal-to-noise ratio [282,283,289]. In bacterial colonies, which contain an extracellular matrix rich in proteins, the use of high-power near-infrared lasers at this wavelength further reduces fluorescence interference while still operating within the high quantum efficiency spectral region of silicon CCDs. On top of that, analyzing entire colonies is easier to prepare and provides more information because the sample incorporates extracellular materials and various metabolic activities within the colonies. Additionally, the cell-to-cell variation often seen in single-cell analysis is minimized, as the signal is averaged across a huge amount of different cells [136].

Moreover, the longer wavelength of the 785 nm fiber probe allows for deeper penetration into biological samples compared to visible and UV lasers. This enhanced penetration is especially beneficial for studying thicker or multilayered samples, such as biofilms, as it enables the collection of Raman signals from depths of approximately hundreds of microns within the sample. Furthermore, near-infrared lasers induce less photochemical degradation of samples as compared to ultraviolet (UV) or visible lasers, allowing higher laser power for the excitation of Raman photons [200,223,281,288,290,291], which increases signal intensity and reduces measurement time.

When comparing the spectra obtained from both UV, visible, and NIR excitations, several notable differences emerge. Specifically, the peak positions, their intensities, and the relative intensities of other signals in the spectrum are not identical between the three methods. These discrepancies may stem from several factors. First, variations in the Raman scattering cross-sections of the different molecules involved can significantly alter the spectra with different excitation wavelengths [136,292]. Second, the samples and their preparation methods differ for the different types of excitations. Additionally, the devices used may exhibit varying CCD sensitivities across different spectral regions, which is expected when employing different excitation wavelengths. Finally, these differences could also be attributed to the pre-processing of the spectra, as the datasets were pre-processed separately with distinct parameters.

## 3. Conclusions

This study emphasizes the critical role of selecting the appropriate excitation wavelength in Raman spectroscopy for bacterial analysis, as UV, visible, and NIR wavelengths each offer distinct advantages and limitations. The optimization of experimental parameters—such as wavelength, spatial resolution, and laser power—is crucial for obtaining reliable and reproducible Raman spectral data.

UV resonance Raman (UVRR) spectroscopy provides strong signal intensity with minimal fluorescence interference, making it particularly effective for detecting specific cellular components like nucleic acids and aromatic amino acids, though the risk of photodamage remains a concern. Visible (VIS) excitation offers high signal intensity and spatial resolution, ideal for single-cell analysis, but is more susceptible to fluorescence interference. Near-infrared (NIR) excitation, such as at 785 nm, produces weaker Raman signals but reduces fluorescence and photodamage while allowing deeper sample penetration, making it suitable for in situ measurements.

The technological differences between these wavelengths highlight the need for careful experimental design. Factors such as laser power, sample characteristics, spatial resolution, fluorescence background, and acquisition time significantly impact spectral quality. Additionally, variations in bacterial growth media, and whether individual cells or colonies are measured, can introduce variability in results.

Beyond the technical considerations, the effective processing of Raman spectra—coupled with the careful application of machine learning algorithms—ensures robust and accurate bacterial identification. Careful algorithm selection prevents issues like overfitting or underfitting, ultimately leading to more reliable results. Integrating optimized experimental design and data analysis is essential for producing meaningful and reproducible outcomes in bacterial studies.

## 4. Materials and Methods

### 4.1. Bacteria Cultivation and Sample Preparation

Six bacterial strains were used in this study. All of them were purchased from the German Collection of Microorganisms and Cell Cultures, Braunschweig (DSMZ). The bacteria investigated were *Escherichia coli* DSM 498, *Klebsiella terrigena* DSM 2687, *Enterococcus maloduratus* DSM 20681, *Pseudomonas stutzeri* DSM 5190, *Staphylococcus cohnii* DSM 20262, and *Staphylococcus warneri* DSM 20036. The bacteria were grown on tryptic soy agar (TSA) and incubated at 37 °C overnight. Briefly, the solid media used in this study were prepared with 17 g/L casein peptone, 3 g/L soya peptone, 5 g/L sodium chloride, 2.5 g/L dipotassium hydrogen phosphate, 2.5 g/L glucose, and 15 g/L agar (Sigma Aldrich, Munich, Germany).

For UVRR, two to three loopfuls of bacterial biomass from the overnight culture were transferred from the TSA plate into 1 mL dH_2_O in two separate Eppendorf tubes. Some bacteria, such as *E. maloduratus*, had grown very poorly, which is why the entirety of the TSA plate was washed with 1 mL dH_2_O. The bacterial suspension was then subjected to three consecutive washing steps with 1 mL dH_2_O using centrifugation at 13,400 rpm for 2 min (Minispin; Eppendorf, Wesseling-Berzdorf, Germany). The bacterial pellet was then resuspended in 300 µL of dH_2_O, and both replicates were air dried at room temperature for 30 min onto fused silica slides.

For Raman microscopy in the VIS region, a loopful of bacterial biomass from the overnight culture was transferred into 1 mL dH_2_O. The bacterial suspension was then washed three consecutive times with dH_2_O as described above and reconstituted into 200 µL dH_2_O. A total of 10 µL of the suspension was placed in tiny droplets onto the nickel foil. Depending on the turbidity of the sample, serial dilutions were employed. Prior to Raman measurements, the suspension droplets were dried at room temperature on the nickel foil.

To reduce the background noise of the conventional Petri dish’s Raman signal on the bacterial spectra for the Raman fiber probe, overnight cultures of the bacteria were incubated on stainless steel Petri dishes (75 mm, Bochem, Weilburg, Germany) at 37 °C containing 8 mL TSA. The bacteria were then directly measured in the Petri dish.

The sample measurement procedures were tailored to each wavelength to optimize data quality and minimize interference. For UVRR, the bacterial sample was analyzed as liquid droplets in high bacterial load (~100 µL) to minimize photodamage. This approach necessitated refocusing the laser after acquiring each spectrum to maintain precision. In contrast, measurements at 532 nm were conducted on samples dried onto nickel foil. This ensured the bacteria remained stationary during measurement and minimized interference from water vibration bands. For the 785 nm wavelength, measurements were performed using a fiber probe within stainless steel agar plates, providing a moisture-rich environment. This setup inherently influenced the Raman spectra by introducing environmental moisture effects.

### 4.2. Raman Spectroscopic Measurements

#### 4.2.1. UV Resonance Raman Spectroscopy

The Raman spectra were obtained with a Raman microscope (HR800; Horiba Jobin-Yvon, Bensheim, Germany) coupled with three different laser systems and an 800 mm focal length for the bulk measurements in the deep UV regions. A 20× antireflection-coated objective (LMU UVB; numerical aperture 0.4) was utilized to channel and focus the laser. A nitrogen-cooled CCD camera caught the backscattered Raman light, which was sent via a 400 µm entrance slit and onto a 2400 line/mm grating. For excitation, three different Toptica laser systems (Toptica, Munich, Germany) were used: 229 nm (TOPWAVE 229-010, 10 mW), 244 nm (DLC TA-FHG PRO, 50 mW), and 257 nm (TOPWAVE 257-015, 15 mW).

The sample was spun at a speed of 30 rpm and moved on a spiral path with a 4 mm diameter to cover a broad sample area in an effort to reduce photodegradation. Teflon was measured to calibrate the wavenumber prior to each measurement. In order to reduce culture artefacts, the measurements were performed in three biological replicates. During each measurement session, all six bacteria were measured, consisting of 60 spectra × 30 s integration.

#### 4.2.2. Raman Microscopy

The Raman spectra were obtained using a frequency doubled Nd:YAG laser (LCM-S-111-NNP25; Laser-export Co., Ltd., Moscow, Russia) linked to a 532 nm excitation wavelength using a Raman microscope BioParticleExplorer (BPE, Rap.ID Particle Systems GmbH, Berlin, Germany). Using a 100× air objective (MPLFLN-BD, NA = 0.90, Olympus, Tokyo, Japan) and a laser power of around 9 mW, the laser beam was focused on the sample with a spot size of less than 1 µm. Per spectrum of a single bacterial cell, the integration time was 10 s. A 920 lines/mm grating was used in conjunction with a single-stage monochromator (HE 532, Horiba Jobin Yvon, Bensheim, Germany). With a spectral resolution of around 8 cm^−1^, the detector was a thermoelectrically cooled charge-coupled device (CCD) camera (DV401-BV; Andor Technology, Belfast, UK).

4-acetamidophenol (4-AAP) was measured to calibrate the wavenumber prior to each measurement. In order to reduce culture artefacts, the measurements were performed in three biological replicates.

#### 4.2.3. Raman Fiber Probe

Using a Raman fiber probe setup (Kaiser Optical Systems, Ann Arbor, MI, USA) and a single-mode diode laser (Toptica, Munich, Germany) excited at 785 nm, Raman spectra from bacterial colonies were obtained. A Raman fiber probe (Inphotonics, Norwood, CO, USA) with a focal spot width of about 50 μm and a depth of field of approximately 200 μm focused the laser light on the sample, providing approximately 200 mW of power to the sample plane with an equivalent irradiance of about 104 W/cm^2^. After passing through a holographic transmissive grating, the scattered Raman signal was detected on an open-electrode charge-coupled device (CCD) chip (Andor, Belfast, UK) that was thermoelectrically cooled and backlit. The system’s spectral resolution is around 4 cm^−1^. The fiber probe was positioned vertically downward towards the Petri dish and fastened to a movable platform. The fiber was aimed towards the microbial colonies until the bacteria’s Raman signal was clearly visible. For daily calibration, 4-AAP was used.

### 4.3. Data Pre-Processing and Multivariate Data Analysis

The pre-processing and subsequent data analysis of the Raman spectra for all excitation wavelengths were performed using the RAMANMETRIX software Version 0.6.0 (https://ramanmetrix.eu/, (accessed on 3 July 2024)) [293]. Prior to the analysis and model creation, several pre-processing steps were performed with some slight variations. The pre-processing contains several steps starting with the removal of spectral artifacts, unwanted signals, and cosmic rays by cutting off below 350 cm^−1^ and above 3150 cm^−1^ [294]. For the despiking of the Raman spectra obtained by the BPE, a two spectra presence analysis was applied, while for the UVRR and NIR the default settings were chosen. The wavenumber calibration on the wavenumber axis was performed with the spectra of Teflon for the UVRR spectra and 4-AAP for the VIS and NIR Raman spectra [295].

Afterwards, UVRR and NIR sample spectra were baseline corrected using a Sensitive Nonlinear Iterative Peak (SNIP) clipping algorithm and vector normalization. The baseline correction for the VIS Raman spectra consisted of a combination of SNIP and extended multiplicative scattering correction (EMSC) with a polynomial degree of 5, which allows for the handling of large variations in fluorescence background without setting large degrees for EMSC [296]. Spectra were then truncated to the relevant range of 350–3150 cm^−1^. Finally, the silent region from 1800 to 2600 cm^−1^ was cropped for the VIS region Raman spectra, while the wavenumber region from 1800 cm^−1^ onward was removed for the UVRR and the NIR [297]. The UVRR and VIS Raman spectra were subjected to quality filters in order to eliminate unwanted outliers. Moreover, the spectral region between 300 and 600 cm^−1^ was excluded for the NIR measurements due to artifact interference from the quartz material of the fiber probe. This exclusion was necessary to avoid misinterpretation of the data and ensure the accuracy of the spectral analysis.

To compare the mean Raman spectra, a combination of principal component analysis (PCA) and linear discriminant analysis (LDA) was used with varying amounts of principal components (PCs) for N-fold cross validation. The number of PCs was adjusted depending on the chosen method and excitation wavelength.

## Figures and Tables

**Table 1:** Tentative band assignment for 229, 244, and 257 nm excitation.

Wavenumber/cm^−1^			Assignment	Reference
229	244	257		
	728	727	Ring breathing modes of adenine	[72,158,163]
764	764		Tryptophan (W18) and phenylalanine: symmetric benzene/pyrrole in-phase ring breathing mode	[82,84,221,224]
	785	787	Ring breathing modes of cytosine, uracil, and thymine	[222]
830			Tyrosine (Y1): ring breathing mode	[79,82,84]
857	854		Tryptophan (W17): ring breathing mode Ring breathing vibration of tyrosine	[79,222,223]
881			Tryptophan (W17)	[79,82]
	956		Tyrosine	[222]
1013	1007	1015	Tryptophan (W18) and phenylalanine: symmetric benzene/pyrrole in-phase ring breathing mode	[82,84,221,224,225]
1181	1178	1177	Tyrosine (Y9a): in-plane CH bending C, T: CC and CN stretching	[82,221,225,226]
1214			Tyrosine (Y7a) and phenylalanine: ring C–C stretching	[82,221,225]
	1253	1249	Amide III	[222]
	1334	1334	Adenosine out-of-phase stretching (C5N7 and C8N7) Guanine and tyrosine	[72]
1340			Tryptophan (W7): Fermi resonance between N1 and C8 stretching in pyrrole ring and combination bands of out-of-plane bending A: C5N7 and N7C8 stretching G	[106,221,224,225]
1361			Tryptophan (W7): Fermi resonance between N1–C8 stretching in pyrrole ring and combination bands of out-of-plane bending G: N7C8, N1C6, and N5N7 stretching	[82,106,221,225]
	1367	1372	Cytosine and thymine	[72,101,230]
	1418	1423	G, A: C4N9 and C8H deformation	[226,231]
1481	1481	1480	G: C8H deformation, and N9C8 and C8N7 stretching A: C8H deformation and N9C8 stretching A, G: stretching along the long axis of the purines	[106,224,226,227]
1520			Tryptophan	[84]
	1532	1534	C: N3=C4 stretching	[72,231]
1553			Tryptophan (W3): C–C stretching vibration of the pyrrole ring Tyrosine (Y8b): in-plane ring stretching	[82,221,225]
	1571	1573	A: C4C5, C4N3 and N6H2 stretching vibrations G: ring vibrations	[231]
1613		1612	Tyrosine (Y8a) and tryptophan (W1): in-plane C=C ring stretching	[82,221,225]
	1640	1648	C: C2 O stretching T: C4 O–C4C5 G: (N2–H2) scissoring	[226,231]
1670			Amide I C=C stretching of lipids	[228,229]
1766			Tryptophan (W18+W16 combination)	[82,106,227]

**Table 2:** Summary of the classification results of the different bacteria measured at 229, 244, and 257 nm.

	229 nm			244 nm			257 nm		
Species	Acc. /%	Sens. /%	Spec. /%	Acc. /%	Sens. /%	Spec. /%	Acc. /%	Sens. /%	Spec./%
*E. coli*	98	99.2	99.1	97.6	100	100	96.1	98.9	99.6
*E. malodoratus*		99.0	99.5		100	100		88.9	99.2
*K. terrigena*		99.2	99.8		99.4	100		99.4	99.8
*P. stutzeri*		95.8	100		100	100		98.5	99.8
*S. cohnii*		97.8	99.5		95.7	98		97.2	99.6
*S. warneri*		97.4	99.8		90.6	99.2		94.4	97.3

Acc.: accuracy; Sens.: sensitivity; Spec.: specificity.

**Table 3:** Summary of the validation results of the different bacteria measured at 229, 244, and 257 nm.

	229 nm			244 nm			257 nm		
Species	Acc. /%	Sens. /%	Spec. /%	Acc. /%	Sens. /%	Spec. /%	Acc. /%	Sens. /%	Spec. /%
*E. coli*	88.9	100	88.2	86.4	100	99	76.7	86.7	97.3
*E. malodoratus*		100	99.7		100	100		18.3	99.7
*K. terrigena*		53.3	100		94.9	100		100	100
*P. stutzeri*		86.7	99.6		100	100		86.7	97.3
*S. cohnii*		98.3	99.6		100	84.8		98.3	78.3
*S. warneri*		100	99.3		25	100		70	99.3

Acc.: accuracy; Sens.: sensitivity; Spec.: specificity.

**Table 4:** Observed Raman bands and their tentative assignments for 532 nm excitation.

Wavenumber/cm^−1^	Assignment	Reference
723	Ring breathing modes of adenine	[136,143,144,148,158,159,163]
749	Cytochrome	[179,180,181,183,184,185,241]
782	Ring breathing modes of cytosine, uracil, and thymine	[143,144,163,222,244]
854	Ring breathing vibration of tyrosine	[143,144,163,170,186,245]
1004	Ring breathing modes of phenylalanine and tryptophan	[82,84,143,148,186,206,221,224,225,235,244,245,246]
1098	PO_2_^−^ symmetrical stretching and C–C and C–O–C skeletal stretching—glycosidic linkage of polysaccharides	[136,170,222]
1126	Cytochrome	[179,180,181,183,184,185,241]
1245	Amide III	[136,143,144,148,229,244,245,246]
1312	Cytochrome	[179,180,181,183,184,185,241]
1333	Ring vibrations of guanine and adenine	[136,143,144,159,170,171,244,247]
1452	CH_2_/CH_3_ deformation vibrations of lipids, proteins, and carbohydrates	[59,136,144,148,170,228,234,235,245,246]
1577	Ring vibrations of guanine and adenine	[143,158,159,163,170,186,207,222,244]
1583	Cytochrome	[179,180,181,183,184,185,241]
1609	C=C ring vibrations of phenylalanine, tyrosine, and tryptophan	[144,222,244,245]
1664	Amide I	[59,136,143,148,207,245,246]
2936	CH_3_/CH_2_ stretching vibrations	[136,144,186,229,236,246]

**Table 5:** Summary of the classification and validation results of the different bacteria measured at 532 nm.

	Classification			Validation		
Species	Acc. /%	Sens./%	Spec. /%	Acc. /%	Sens. /%	Spec. /%
*E. coli*	95.0	96.3	98.3	89.3	100	95.6
*E. malodoratus*		100	99.3		100	99.6
*K. terrigena*		88.9	98.7		80.0	92.4
*P. stutzeri*		98.1	100		97.8	100
*S. cohnii*		95.6	98.4		96.2	99.2
*S. warneri*		91.3	99.1		61.5	100

Acc.: accuracy; Sens.: sensitivity; Spec.: specificity.

**Table 6:** Observed Raman bands and their tentative assignments for 785 nm excitation.

Wavenumber/cm^−1^	Assignment	Reference
725	Ring breathing modes of adenine	[144,202,205,206,213,252,253,254,255,256]
779	Ring breathing modes of cytosine, uracil, and thymine	[144,199,202,205,206,252,253,254,256,257,258]
847	Ring breathing vibration of tyrosine	[143,144,199,202,205,206,256,257,258,259]
1004	Ring breathing modes of phenylalanine and tryptophan	[136,143,144,199,202,206,212,252,256,257,258,259,260]
1085	PO_2_^–^ symmetrical stretching and C–C and C–O–C skeletal stretching—glycosidic linkage	[144,202,205,206,213,252,253,254,255,256]
1127	Stretching vibrations of C–O–C glycosidic linkage, and C–C and C–N skeletal stretching acyl (trans conformation) of lipids	[143,144,199,202,205,206,212,213,252,253,256,259]
1208	Stretching vibrations of C–C_6_H_5_ in phenylalanine, tyrosine, and tryptophan	[144,205,212,213]
1250	Amide III	[136,144,202,206,212,252,253,254,256,259,260,261]
1336	Ring vibrations of guanine and adenine	[144,205,206,252,254,256,258,260,262,263]
1451	CH_2_/CH_3_ deformation of lipids, proteins, and carbohydrates	[144,199,202,205,206,213,252,254,256,257,258,262]
1581	Ring vibrations of guanine and adenine	[143,199,202,205,206,212,213,258]
1609	C=C ring vibrations of phenylalanine and tyrosine	[136,144,199,202,256]
1659	Amide I	[136,143,144,199,202,205,206,212,252,253,256,258,260,261,262,264]

**Table 7:** Summary of the validation results of the different bacteria measured at 785 nm.

	Classification			Validation		
Species	Acc. /%	Sens. /%	Spec. /%	Acc. /%	Sens. /%	Spec. /%
*E. coli*	98.7	100	99.7	95.2	100	99.6
*E. malodoratus*		100	99.7		100	100
*K. terrigena*		100	100		100	100
*P. stutzeri*		93.8	99.4		97.7	100
*S. cohnii*		98.4	99.7		93.3	96.0
*S. warneri*		98.2	99.7		80.0	98.7

Acc.: accuracy; Sens.: sensitivity; Spec.: specificity.

## Data Availability

Data will be made available on request.
